# Influence of 7T GRE-MRI Signal Compartment Model Choice on Tissue Parameters

**DOI:** 10.3389/fnins.2020.00271

**Published:** 2020-04-28

**Authors:** Kiran Thapaliya, Viktor Vegh, Steffen Bollmann, Markus Barth

**Affiliations:** ^1^Centre for Advanced Imaging, The University of Queensland, Brisbane, QLD, Australia; ^2^ARC Centre for Innovation in Biomedical Imaging Technology, Brisbane, QLD, Australia; ^3^School of Information Technology and Electrical Engineering, The University of Queensland, Brisbane, QLD, Australia

**Keywords:** myelin imaging, frequency shift, phase unwrapping, white matter, corpus callosum, signal compartmentalization

## Abstract

Quantitative assessment of tissue microstructure is important in studying human brain diseases and disorders. Ultra-high field magnetic resonance imaging (MRI) data obtained using a multi-echo gradient echo sequence have been shown to contain information on myelin, axonal, and extracellular compartments in tissue. Quantitative assessment of water fraction, relaxation time (T_2_*), and frequency shift using multi-compartment models has been shown to be useful in studying white matter properties *via* specific tissue parameters. It remains unclear how tissue parameters vary with model selection based on 7T multiple echo time gradient-recalled echo (GRE) MRI data. We applied existing signal compartment models to the corpus callosum and investigated whether a three-compartment model can be reduced to two compartments and still resolve white matter parameters [i.e., myelin water fraction (MWF) and g-ratio]. We show that MWF should be computed using a three-compartment model in the corpus callosum, and the g-ratios obtained using three compartment models are consistent with previous reports. We provide results for other parameters, such as signal compartment frequency shifts.

## Introduction

Myelin is one of the main components of the white matter tissue in the brain that acts as an axonal insulator to help conduct neuronal signals (Caminiti et al., 2009). The demyelination has been associated with different white matter diseases like multiple sclerosis, schizophrenia, brain stroke, and even Alzheimer’s disease (Moore et al., 2000; Laule et al., 2004; MacKay et al., 2006; Bejanin et al., 2016; Lehto et al., 2016). Different methods based on magnetic resonance (MR) such as T_1_ and T_2_ relaxation (McDESPOT) (Deoni et al., 2011), diffusion tensor imaging (Billiet et al., 2015; Davies-Thompson et al., 2016), magnetization transfer ratio (Grossman et al., 1994; Schmierer et al., 2004), ultra-short echo time (UTE) (Horch et al., 2011; Wilhelm et al., 2012), and T_1_-weighted/T_2_-weighted image ratio mapping methods have been used as a sensitive biomarker for myelin in multiple sclerosis and schizophrenia (Beer et al., 2016; Granberg et al., 2017) and to visualize that myelin contrast in the brain (Ganzetti et al., 2014).

Methods based on the spin-spin relaxation time (i.e., T_2_) mapping (Mackay et al., 1994; Whittall et al., 1997) using the multi-echo spin echo sequence was used to estimate myelin water fraction (MWF) in the white matter. The mathematical modeling technique used for signal compartmentalization was based on the non-exponentially decaying T_2_ curve acquired using a multi-echo spin echo sequence. This technique dissociates signals from different tissue compartments (myelin, axonal, or extracellular), wherein the shortest T_2_ signal compartment is assumed to be myelin water (Mackay et al., 1994; Lancaster et al., 2003). The myelin parameters have been extracted from multi-echo spin echo magnitude data (Mackay et al., 1994; Laule et al., 2004). The approach has been used to study different brain diseases, such as multiple sclerosis and schizophrenia, wherein a statistically significant reduction in MWF was present (Flynn et al., 2003; Wright et al., 2016).

However, multi-echo spin echo sequence produces higher specific absorption rates in tissue due to the use of multiple refocusing 180° pulses. The spin echo data also have a higher sensitivity to radio frequency (RF) field inhomogeneities, and generally, a smaller volume coverage with lower resolution can be achieved. Alternatively, the multi-echo gradient-recalled echo (GRE) magnetic resonance imaging (MRI) sequence with a single RF pulse with low flip angles (FAs) can provide an alternative data collection strategy which overcomes drawbacks associated with the spin echo sequence. Since the GRE-MRI data are collected in the T_2_* regime, different tissue compartment models need to be developed.

A number of signal compartment models for GRE-MRI data have been proposed for the mapping of various tissue parameters (Hwang et al., 2010; Sati et al., 2013; Nam et al., 2015; Thapaliya et al., 2018). The models consist of the myelin, axonal, and extracellular signal compartments, and each compartment has a water fraction, T_2_^∗^ value, and frequency shift. Note that GRE-MRI data consist of both magnitude and phase information. Researchers have demonstrated white matter orientation dependence with respect to the scanner field in the mapped frequency shift value (Wharton and Bowtell, 2012), alongside shape and size of fibers (Xu et al., 2017) and their packing (Chen et al., 2013). Notably, the myelin sheath is a layered structure which insulates axons, and it is comprised of lipids (∼70%) and proteins (∼30%) (Morell and Quarles, 1999). The lipid component has greater influence on GRE-MRI signal phase than the protein component (Lee et al., 2012; Duyn and Schenck, 2016). As such, the GRE-MRI complex voxel signal contains important information on tissue organization and composition from which specific tissue parameters are aimed to be extracted using multi-compartment modeling approaches.

Five different GRE-MRI signal compartment models have been developed to study tissue properties, each having a different number of model parameters. The signal compartment model proposed by Hwang et al. (2010) assumes three water pools, and compartments are characterized by water fraction and relaxation time parameters. This model has seven parameters and can be extracted by fitting GRE-MRI signal magnitude. The use of this model results in large residuals, especially when white matter fiber orientation is perpendicular to the scanner field. Later work demonstrated a reduction in residuals by incorporating frequency shift terms for each compartment, leading to a nine-parameter model, and fitting complex valued signals (Sati et al., 2013). The main challenge of using this model is the removal of macroscopic field effects present in phase images (e.g., phase changes due to air–tissue interfaces, metal–tissue boundaries) which hinder the computation of compartment frequency shifts. While several methods have been proposed to remove macroscopic effects, residual effects remained in the phase images (Neelavalli et al., 2009; Liu et al., 2011). Instead of correcting for macroscopic field effects in the raw signal, Nam et al. (2015) incorporated the macroscopic field effect into the signal equation, resulting in a 10-parameter model. We extended the model to 11 parameters in prior work, where the additional parameter was used to account for the noise floor in the GRE-MRI data and applied it to regions of the corpus callosum (Thapaliya et al., 2018). Model variants have been used to study lesions in relapsing-remitting multiple sclerosis patients (Li et al., 2015) and dysplastic tissue regions in focal epilepsy (Thapaliya et al., 2019).

Based on the GRE-MRI signal compartment models published previously, a number of points remain unclear: (i) how are tissue parameters influenced by model choice, (ii) how many compartments and parameters are necessary to obtain a robust measure of tissue parameters and g-ratio, and (iii) can specific parameters be the same across different compartments to reduce model complexity?

## Materials and Methods

The study was approved by the university human ethics committee, and written informed consent was obtained from 10 healthy participants (aged 30–41 years). Data were acquired using a three-dimensional (3D) GRE-MRI sequence on a 7T whole-body MRI research scanner (Siemens Healthcare, Erlangen, Germany) with a 32-channel head coil (Nova Medical, Wilmington, DE, United States) using the following sequence parameters: acceleration factor = GRAPPA 2, echo times from 2.04 to 46.41 ms with echo spacing of 1.53 ms (30 echoes in total), repetition time (TR) = 51 ms, FA = 20°, voxel size = 1 × 1 × 1 mm^3^, matrix size = 210 × 168 × 144, and total acquisition time was 6 min 13 s. Individual channel data were processed by computing a signal phase noise map by taking the voxel-wise phase difference between any two channels. The noise map was smoothed using Gaussian filtering, the variance over a 3 by 3 spatial grid was computed and thresholded to form a mask where this two-channel information was averaged. This process was repeated for all two-channel combinations and summed to produce the combined signal phase images (Bollmann et al., 2015). A brain mask for each participant was created using FSL BET (Smith et al., 2004). Phase unwrapping and background field processing were performed using iHARPERELLA^1^ (STI Suite) (Li et al., 2014) to create tissue phase. We use default parameters [number of iterations = 100; padsize = (100 100 100)] in iHARPERELLA to process the acquired data set.

### Region-of-Interest Analysis

**FIGURE 1:**
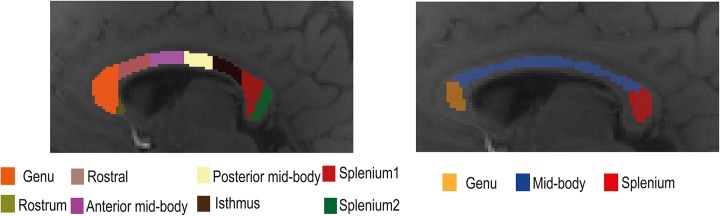
Illustration of the location of the eight **(left)** and three **(right)** regions of interest (ROIs) in the mid-sagittal plane of the corpus callosum used in the gradient-recalled echo (GRE)-MRI signal compartment model analysis.

The corpus callosum was segmented manually into eight regions of interest (ROIs: rostrum, genu, anterior mid-body, posterior mid-body, isthmus, and two splenium regions; see Figure 1) using a standardized template (Witelson, 1989) and with the aid of the MIPAV software^2^ (McAuliffe et al., 2001) using only the magnitude data from individual participants. The template contained eight ROIs, but we only used seven of them. Due to the small number of voxels in the rostrum, this region was omitted from the analyses. For additional analyses, the corpus callosum was also segmented into three primary subregions (genu, mid-body, and splenium) with each region including at least 30 and a maximum of 98 voxels. Data from both the seven and three corpus callosum subregions were used to study tissue parameter changes with model selection over the corpus callosum. White matter fiber orientation in each ROI was assumed to be perpendicular to the scanner magnetic field. During data acquisition, care was taken to orient the line joining the ears of each participant perpendicular to the bore of the scanner. A representative signal for each ROI was obtained by averaging the complex signals from the adjacent slices. Signals from three adjacent slices were averaged to improve signal quality.

### Gradient-Recalled Echo-MRI Signal Compartment Models

As the model described by Thapaliya et al. (2018) is the most general, we outline it and state how other models evaluated differ with respect to it:

(1) $$S\left(t\right)=[A_{\text{my}}e^{-\left(\frac{1}{T_{2,\text{my}}^{{}^{*}}}+i\,2\,\pi \,\Delta \,f_{\text{my}}\right)\,t}+A_{\text{ax}}e^{-\left(\frac{1}{T_{2,\text{ax}}^{{}^{*}}}+i\,2\,\pi \,\Delta \,f_{\text{ax}}\right)\,t}+A_{e\,x}e^{-\left(\frac{1}{T_{2,\text{ex}}^{{}^{*}}}+i\,2\,\pi \,\Delta \,f_{\text{ex}}\right)\,t}+C]e{}^{-i\,2\,\pi \,\Delta \,f_{b\,g}\,t}$$

where *S* represents the complex-valued GRE-MRI signal for a particular ROI or voxel at echo time *t* (in seconds), *A*_my_, *A*_ax_, and *A*_ex_ (in a.u.) represent water fractions for the myelin (my), axonal (ax), and extracellular (ex) compartments, and correspondingly T_2__,my_^∗^, T_2__,ax_^∗^, and T_2__,ex_^∗^ (in seconds), and Δf_my_, Δf_ax_, and Δf_ex_ (in Hz) are the compartment relaxation times and frequency shifts. Additionally, *C* (in a.u.) is used to adjust for the noise floor in the data, and Δf_bg_ (in Hz) is the background frequency shift term used to cater for residual macroscopic field effects in the data. Table 1 summarizes model variants according to descriptions by Sati et al. (2013); Nam et al. (2015), Thapaliya et al. (2018), and two modified models which use parameter reduction to reduce model complexity. Myelin compartment T_2_* value in the 3COMP (three-compartment seven-parameter model) and 2COMP (two-compartment five-parameter model—combining intra and extracellular signals into a single compartment) models was fixed to 7 ms based on previous findings (Thapaliya et al., 2018). We have not considered the model by Hwang et al. (2010) as it used GRE-MRI signal magnitude alone (i.e., Δf_my_ = Δf_ax_ = Δf_ex_ = 0), and it has been shown not to fit the GRE-MRI data as well as the other models did.

**TABLE 1:** Summary of models investigated and parameter settings used for each model indicating where values were either fixed or free (i.e., X).

**Model**	***P***	***A*_my_**	***A*_ax_**	***A*_ex_**	*T*_2__,my_*	***T*_2__,ax_^∗^**	***T*_2__,ex_^∗^**	**Δ*f*_my_**	**Δ*f*_ax_**	**Δ*f*_ex_**	**Δ*f*_bg_**	***C***
Thapaliya	11	X	X	X	X	X	X	X	X	X	X	X
Nam	10	X	X	X	X	X	X	X	X	X	X	0
Sati	9	X	X	X	X	X	X	X	X	X	0	0
3COMP	7	X	X	X	7	X	*T*_2_,_2_^∗^	X	X	X	0	0
2COMP	5	X	X	0	7	X	0	X	X	0	0	0

P represents the total number of free model parameters for a particular model, fixed T_2_* values are in ms, Δ*f* are in Hz, and *C* has arbitrary units.

Signal magnitude and background field-corrected phase images generated for each echo time were used to form echo time-dependent complex voxel signals. Voxel signals were averaged in ROIs to form region-specific temporal complex valued GRE-MRI signals. For voxel-wise analyses, complex-valued voxel signals were smoothed using the method of robust smoothing and by setting the smoothing kernel equal to 0.01 prior to voxel-wise analysis (Garcia, 2010). Model parameters were estimated using non-linear fitting in MATLAB^®^ for both the region-specific and voxel-level signals. Table 2 provides the initial values, and lower and upper bounds set in the optimization.

**TABLE 2:** Conditions used for the optimization of model parameters.

	***A*_my_**	***A*_ax_**	***A*_ex_**	*T*_2_*	**Δ*f*_my_**	**Δ*f*_ax_**	**Δ*f*_ex_**	**Δ*f*_bg_**	***C***
IV	0.1| *S*(*t*_1_)|	0.6| *S*(*t*_1_)|	0.3| *S*(*t*_1_)|	48	30	−2	5	0	0
LB	0	0	0	0	−200	−50	−50	−20	0
UB	2| *S*(*t*_1_)|	2| *S*(*t*_1_)|	2 | *S*(*t*_1_)|	200	200	50	50	20	0.3| S(*t*_1_)|

T_2_* value in ms, Δ*f* in Hz, and *C* in arbitrary units. *S* represents the signal, and *t*_1_ corresponds to the first echo time. IV, initial value; LB, lower bound; UB, upper bound.

The myelin water fraction was computed using the ratio of the signal amplitude of the myelin water compartment with respect to the total signal [i.e., MWF = *A*_my_/(*A*_my_ + *A*_ax_ + *A*_ex_)]. Axonal water fraction (AWF) and extracellular water fraction were calculated in a similar manner.

### Estimation of the g-Ratio

The g-ratio is a proxy for myelination and measures the ratio of inner axon diameter over outer axon diameter (i.e., including the myelin sheath). A value close to 1 suggests loss of myelin, and values less than 1 reflect mean myelination levels. The g-ratio was calculated according to an established method (Stikov et al., 2015):

$$g-r\,a\,t\,i\,o=\sqrt{\frac{1}{1+\frac{k_{a}\,M\,W\,F}{k_{m}\,A\,W\,F}}},$$

where MWF and AWF are the myelin and axonal water fractions obtained using the compartment models, *k*_*a*_ and *k*_*m*_ are scalars used to adjust between water fractions and volume fractions, and *k*_*a*_ = 0.85 and *k*_*m*_ = 0.4 (Jung et al., 2016). For the sake of comparison, and to benchmark methods, we also provide previously published g-ratios in the corpus callosum (Sati et al., 2013; Nam et al., 2015; Thapaliya et al., 2018).

### Model Selection

Model selection was performed using the corrected version of the Akaike information criterion (Burnham and Anderson, 2004) defined as:

$$A\,I\,C_{c}=N\,\log\left(\frac{R\,S\,S}{N}\right)+2\,P+\underset{\text{CORRECTION}}{\underbrace{\frac{2\,P\,\left(P+1\right)}{N-P-1}}},$$

where *N* is the number of data points used to generate the metric, *RSS* is the residual sum of squares, *P* is the number of free parameters (see Table 1 for the various models), and the correction term is required when *N*/*P* < 40, and in our case, it was always applied. For each model, an AIC_c_ value is generated, and the model with the smallest value is the most parsimonious choice.

## Results

### Region-Based Model Analysis

**FIGURE 2:**
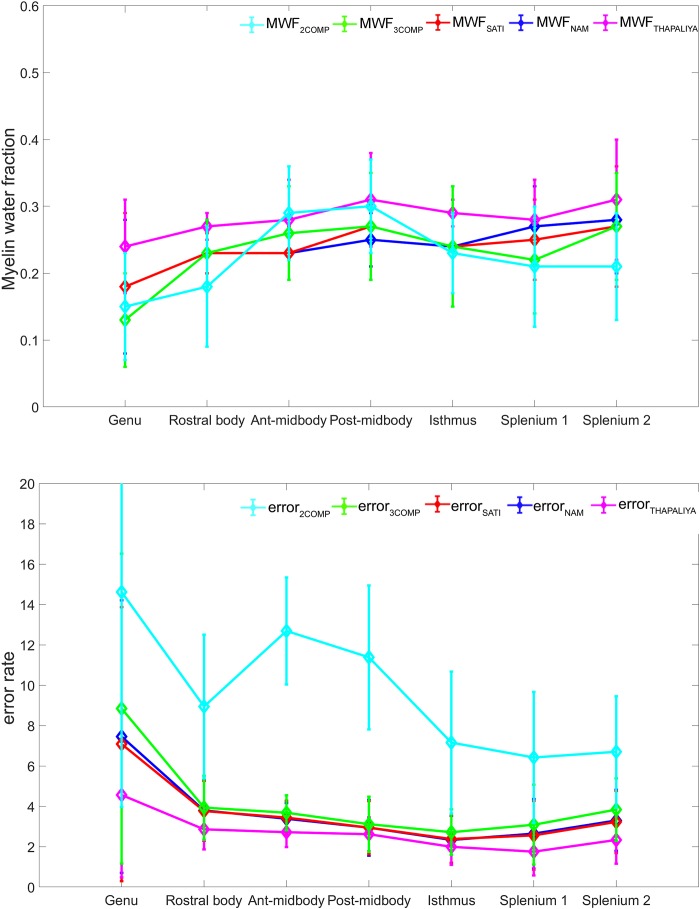
Myelin water fraction (MWF) results for corpus callosum regions of interest (ROIs) **(top)** and model fitting error for the different models **(bottom)**. The mean and standard deviations across participants are provided.

**FIGURE 3:**
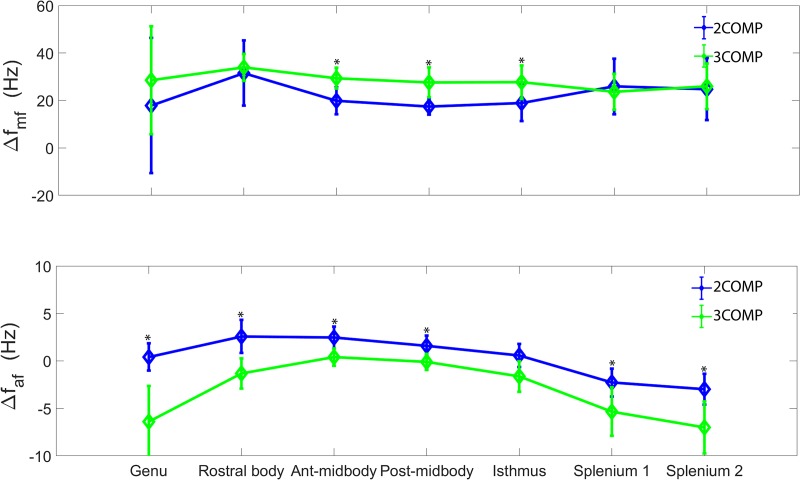
Myelin (Δ*f*_my_), and axonal (Δ*f*_ax_) frequency shifts generated using 3COMP and 2COMP models over the seven corpus callosum regions. * represents the significant differences.

Our investigation primarily focused on MWF and g-ratio. Figure 2 provides the MWF calculated using the various GRE-MRI signal compartment models (refer to Table 1) and the fitting error associated with each of the models. The 2COMP model produced the largest variation in the mean MWF value across the corpus callosum ROIs, and it also has the largest inter-participant variation. The 2COMP model leads to around a factor of two greater fitting error than the other methods. The 3COMP, Sati, Nam, and Thapaliya methods led to fairly consistent MWF values with similar fitting errors. A significant difference between 3COMP and 2COMP in the MWF parameter was found in the posterior mid-body of the corpus callosum (*p* = 0.02). The largest difference between two models was in the axonal frequency shift (*p* < 0.005) across all corpus callosum regions, and significant differences in myelin frequency shift were also present at the mid-body regions of the corpus callosum (*p* < 0.02) (Figure 3). All the tissue parameters estimated from different modelling techniques are provided as Supplementary Tables S1ߝS7.

AIC_c_ values are provided in Table 3. Three-compartment models (3COMP, Sati, Nam, and Thapaliya) outperformed the two-compartment model (2COMP) for all seven smaller regions, the three larger regions, and for the whole of the corpus callosum, irrespective of the number of free model parameters. The three-compartment models performed similarly, and different models were better suited for different regions. Based on the whole corpus callosum, the 3COMP model can suitably capture the trend in the GRE-MRI complex signal. These findings suggest that the 2COMP model is inadequate and, hence, will be omitted from subsequent region-based findings.

**TABLE 3:** List of Akaike information criterion (AIC_c_) values obtained for the different models.

**Regions**	**3COMP**	**Sati**	**Nam**	**Thapaliya**	**2COMP**
Genu	196	190	195	178	239
Rostral body	167	176	181	173	205
Anterior mid-body	179	179	183	170	235
Posterior mid-body	163	168	172	166	220
Isthmus	147	142	146	143	184
Splenium1	148	142	149	129	191
Splenium2	171	142	168	147	193
Genu	213	218	223	214	241
Mid-body	176	176	180	177	193
Splenium	168	173	180	178	197
Whole CC	180	181	185	189	195

*Results have been organized to show that all of the three-compartment models with different number of parameters (3COMP, Sati, Nam, and Thapaliya) can better capture the trend in the underlying signal than the two-compartment model (2COMP). Results provided are for the seven smaller regions, three larger regions, and for the whole of the corpus callosum (CC).*

**FIGURE 4:**
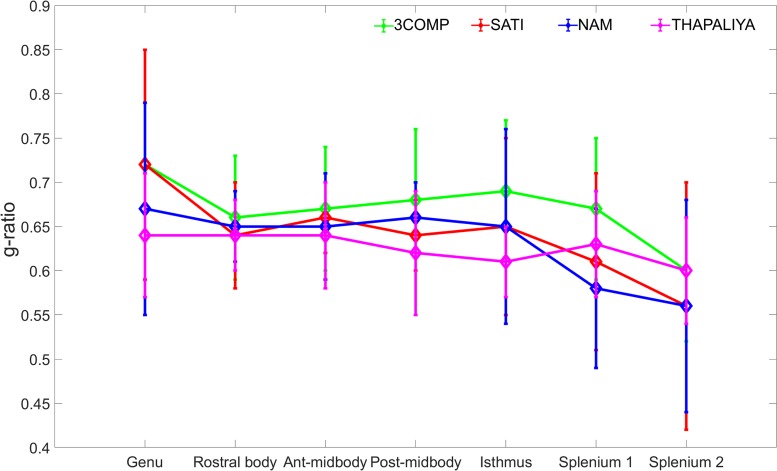
Illustration of g-ratio variations calculated using the myelin water fraction (MWF) and axonal water fraction (AWF) model parameters across the corpus callosum using the various three-compartment models. Mean values were calculated based on data from 10 participants, and error bars represent inter-participant standard deviations.

The g-ratio results are provided in Figure 4. The g-ratio estimated from 3COMP, Sati, Nam, and Thapaliya showed similar values in rostral body and anterior mid-body of the corpus callosum. The g-ratio estimated from 3COMP yielded higher values in comparison to other models in posterior mid-body, isthmus, and splenium1 of the corpus callosum. The g-ratio estimated from Sati, Nam, and Thapaliya showed similar values across the subregions of the corpus callosum. The estimation of the g-ratio from Thapaliya and 3COMP significantly varies at the posterior mid-body and isthmus of the corpus callosum (*p* < 0.01). Comparison of corpus callosum g-ratios obtained across different studies is summarized in Table 4. It can be concluded from this table that g-ratios generated using any of the three-compartment models are consistent with those previously reported in the literature, and humans generally have smaller g-ratios than other species listed.

**TABLE 4:** Summary of g-ratios calculated using the methods assessed here and those published previously for the corpus callosum in different species and using different modalities.

**Method**	**g-ratio**	**Subject**	**Device**
3COMP	0.67 ± 0.08	Human	7T MRI
Sati^1^	0.64 ± 0.08	Human	7T MRI
Nam^2^	0.63 ± 0.08	Human	7T MRI
Thapaliya^3^	0.63 ± 0.05	Human	7T MRI
Mohammadi^4^	0.68 ± 0.05	Human	3T MRI
Stikov^5^	0.70 ± 0.04	Macaque monkey	3T MRI
Mason^6^	0.80 ± 0.01	Mice	EM
Benninger^7^	0.75 ± 0.01	Mice	EM
Arnett^8^	0.76 ± 0.07	Mice	EM
Waxman^9,#^	0.70 ± 0.05	Rabbit	EM

*^1^Sati et al. (2013); ^2^Nam et al. (2015); ^3^Thapaliya et al. (2018); ^4^Mohammadi et al. (2015); ^5^Stikov et al. (2015); ^6^Mason et al. (2001); ^7^Benninger et al. (2006); ^8^Arnett et al. (2001); ^9^Waxman and Swadlow (1976); # implies only the splenium of the corpus callosum; EM, electron microscopy.*

### Voxel-Level Maps

**FIGURE 5:**
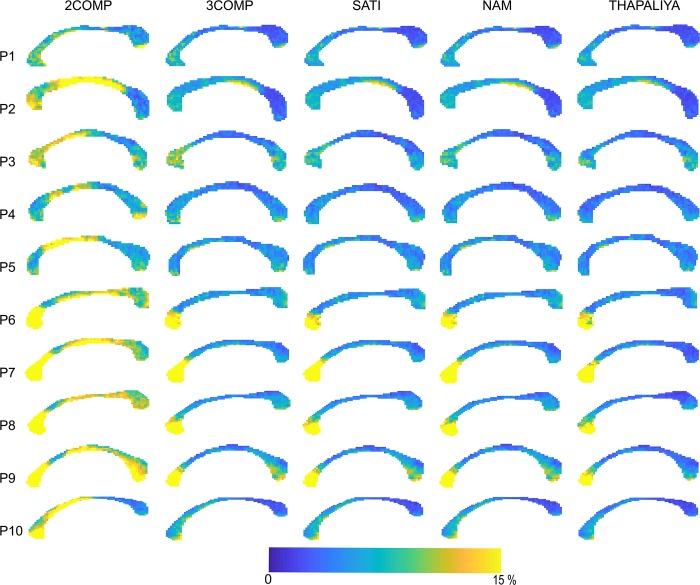
Maps of the model fitting error for the models considered. The fitting error is larger for the two-signal compartment model (2COMP) model, which has two compartments, in comparison with all three-compartment models (3COMP, Sati, Nam, and Thapaliya).

**FIGURE 6:**
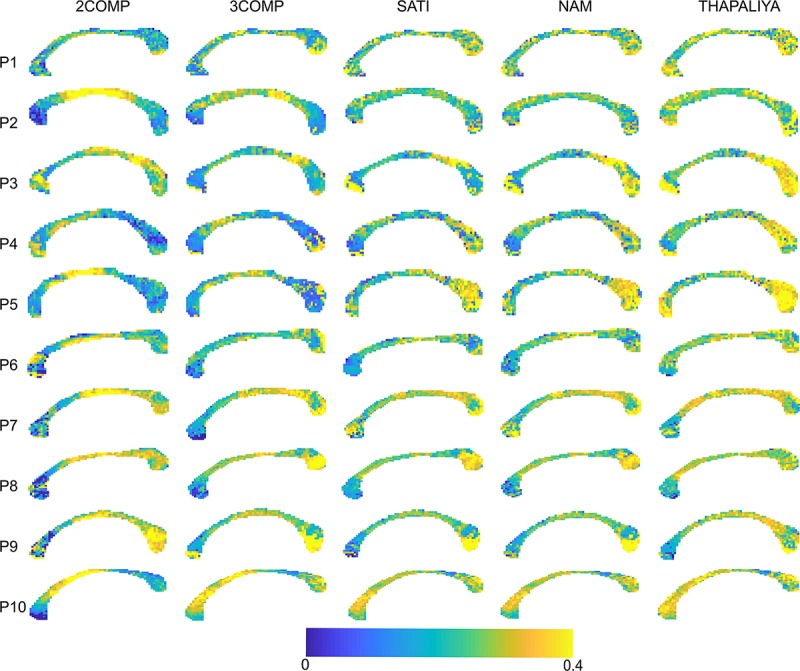
Myelin water fraction (MWF) maps obtained using the two-signal compartment model (2COMP) and three-signal compartment models (3COMP, Sati, Nam, and Thapaliya).

Results in Figure 5 confirm that the model fitting error is larger using two-signal compartments (2COMP) versus three-signal compartments (3COMP, Sati, Nam, and Thapaliya). Figure 6 shows the voxel-by-voxel fit results for MWF for all models in the 10 participants. While our AIC_*c*_ findings from Table 3 indicate that three-comparment models are better suited than the 2COMP model, the model selection process is unable to highlight differences in parameters. As such, percentage difference in the 2COMP MWF parameter with respect to the 3COMP model is provided as Supplementary Figure S1. The result shows that as much as 50% difference in the MWF parameter can be present when the 2COMP model is used. Additionally, Figure 6 suggests that MWF values tend to be larger around the mid-body of the corpus callosum and smaller at the posterior and anterior regions. In some participants, the MWF parameter is also large at the splenium of the corpus callosum. MWF results from Sati, Nam, and Thapaliya are consistent, and some variations are present in the 3COMP results. The axonal and extracellular water fraction maps estimated from different models are provided as Supplementary Figures S2, S3.

Frequency shift parameter results have been provided as Supplementary Figures S4–S6. Pithily, axonal, and extracellular frequency shifts tend to be larger around the mid-body regions, and extracellular frequency shifts are generally larger in amplitude than axonal frequency shift. Myelin frequency shifts tend to be larger at the posterior of the corpus callosum and smaller toward anterior regions.

**FIGURE 7:**
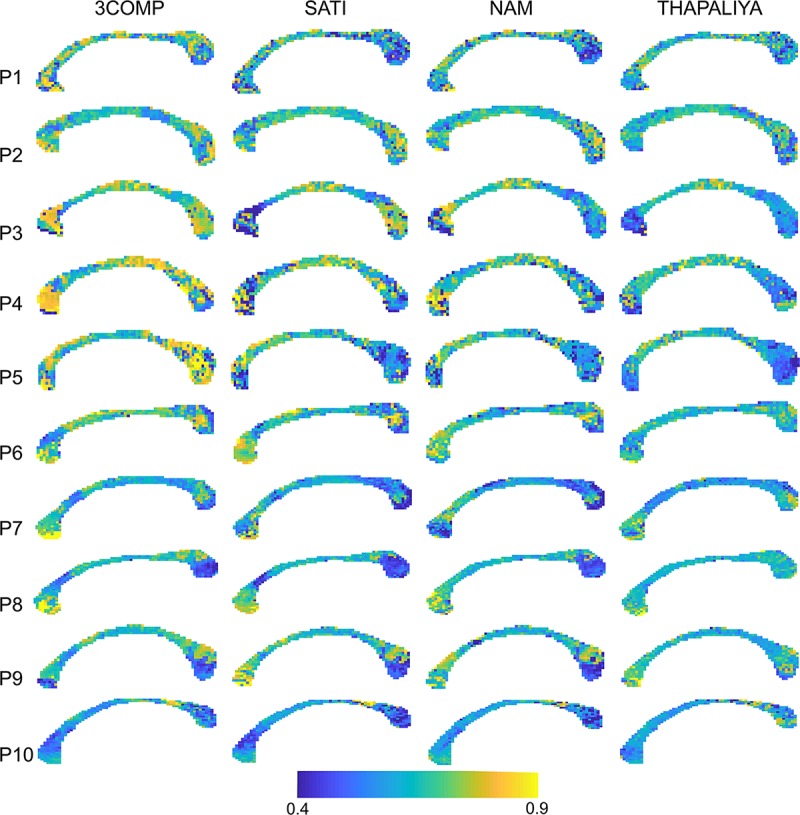
Maps of g-ratio over the corpus callosum estimated using three-signal compartment models (3COMP, Sati, Nam, and Thapaliya) in the 10 participants (P1–P10).

Figure 7 presents voxel-level g-ratio findings across the corpus callosum. The g-ratio estimated using parameters from the 3COMP model produced higher values (especially for participants 3–5) in genu and splenium of the corpus callosum but similar at the middle of the corpus callosum across all participants and in view of the Sati, Nam, and Thapaliya models. The g-ratio is generally in the range 0.5–0.75; however, some voxels have values around 0.4 and few were greater than 0.8. Extensive variations in g-ratios are potentially due to lower fit quality due to noise in the GRE-MRI signal.

### Frequency Shift Distributions Across the Corpus Callosum

**FIGURE 8:**
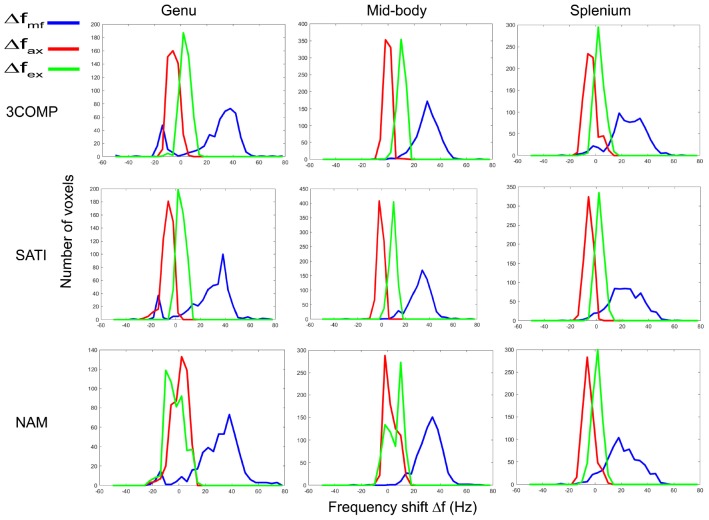
Myelin (Δfmf), axonal (Δfax) and extracellular (Δfex) frequency shift distributions for calculated in three corpus callosum sub-regions. Results provided are for only the three signal compartment models (3COMP, SATI, and NAM).

In this section, we show how frequency shift distributions generated from data from the three-compartment models (3COMP, Sati, and Nam) vary across the three subregions of the corpus callosum. Figure 8 provides the frequency shift distributions for the myelin, axonal, and extracellular signal compartments. Myelin frequency shifts are generally larger and separate out from the axonal and extracellular frequency shifts. A larger level of overlap exists between axonal and extracellular frequency shifts.

## Discussion

We used multi-echo GRE-MRI data to generate signal compartment model parameters based on five different models, each of which having different number of free parameters (5, 7, 9, 10, and 11; Table 1). We found the two-signal compartment model (five-parameter) was unable to describe the signal adequately. All of the three-signal compartment models outperformed the two-signal compartment model, both in region and voxel-based analyses. The Sati (nine-parameter), Nam (10-parameter), and Thapaliya (11-parameter) models all produced slightly better MWF and g-ratio results in comparison with 3COMP (seven-parameter) model, wherein myelin T_2_* value is fixed and axonal and extracellular T_2_* values are assumed to be the same.

*In vivo* human and animal studies have shown that axonal diameter is larger at the middle of the corpus callosum and lower at both ends (Aboitiz et al., 1992a; Alexander et al., 2010; Zhang et al., 2011). These findings have been supported by *ex vivo* histological studies (Aboitiz et al., 1992a; Sepehrband et al., 2016). Myelination and thereby the g-ratio indirectly reflect axon conduction velocity (Tomasi et al., 2012), and axonal density might also affect the frequency shift parameter and tends to be lower at the mid-body and higher at the corpus callosum ends (Schneider et al., 2012). Therefore, GRE-MRI signal compartment modeling sensitive to white matter properties can provide key insight into not only normal aging but also white matter changes due to neurological diseases and disorders.

### Region-Based Findings

Myelin water fractions consistent with our findings (Figure 2) have been reported for the genu, splenium, and across primary corpus callosum regions (Whittall et al., 1997; Liu et al., 2010; Guo et al., 2013; Thapaliya et al., 2018). Most recently, Alonso-Ortiz et al. (2018) used GRE-MRI data to estimate MWF in the genu and splenium of the corpus callosum. An MWF of 14 ± 3% was found in the genu. We found MWF to be 13 ± 7% (3COMP), 18 ± 11% (Sati), 18 ± 10% (Nam), and 25 ± 7% (Thapaliya). In the splenium, Alonso-Ortiz et al. reported 18 ± 7% for MWF, and we found 22 ± 8% (3COMP), 25 ± 6% (Sati), 27 ± 6% (Nam), and 29 ± 7% (Thapaliya) for the splenium1 region, and 27 ± 8% (3COMP), 27 ± 9% (Sati), 28 ± 8% (Nam), and 30 ± 9% (Thapaliya) for the splenium2 region. Values reported here are slightly different to those reported by Thapaliya et al. (2018), which can be attributed to different ROI sizes (larger ROIs in this study). Myelin frequency shift of 3COMP (23.59 ± 9.48 Hz) is similar to that of Li et al. (2015) for the splenium1 of the corpus callosum and also 3COMP (21.31 ± 11.16 Hz) is similar to that of Thapaliya et al. (2018) for the splenium2 of the corpus callosum.

### Voxel-Wise Mapping

The MWF and frequency shift results showed some intersubject variability, and negative myelin frequency shift in some voxels was also estimated by both models as previously reported (Thapaliya et al., 2018). This could be due to the macroscopic field homogeneity which becomes more pronounced in voxel-level analysis, wherein low signal-to-noise ratios are present in comparison with region-based analysis (Nam et al., 2015). Another reason for intersubject variability could be due to the differences in the fiber composition of individuals (Aboitiz et al., 1996). In addition, frequency shifts have been shown to be affected by fiber orientation and compactness (Wharton and Bowtell, 2012); MWF differences could be due to the variation in axonal diameter in fiber bundles (Schneider et al., 2012) and fiber counts which change with aging (Aboitiz et al., 1996). Our number of participants did not allow us to consider the effects of age and gender on MWFs, which in fact vary between males and females (Liu et al., 2010), ultimately affecting g-ratio.

### g-Ratio

Larger g-ratios estimated at the center of the corpus callosum (Figure 7) are similar to those reported in a different study (Mohammadi et al., 2015). The mid-body large values appear to reflect a larger mean axon diameter (Aboitiz et al., 1992b; Schneider et al., 2012). In healthy cat tissue, it was also shown that an increase in g-ratio corresponds to an increase in axon diameter (Berthold et al., 1983). In our study, we did not adjust for brain volume, and it has been established that larger white matter fiber bundles occur in larger brains (Olivares et al., 2001), and g-ratio increases with fiber diameter (Guy et al., 1989). Electron microscopy studies conducted on mice and rabbits additionally imply that g-ratio values could vary across the corpus callosum (Waxman and Swadlow, 1976; Arnett et al., 2001; Mason et al., 2001; Benninger et al., 2006). While our results are slightly different to those reported by others (refer to Table 4), differences may arise from the amount of fibers projecting to different cortical regions and cortical region size with respect to species-specific brain function (Bonzano et al., 2008; Hofer et al., 2008).

### Frequency Shift Distributions

It has been reported that variations in frequency shifts across the subregions of the corpus callosum could be due to the variation of axonal geometry (Björnholm et al., 2017) and mean axonal diameter in fiber bundles (Schneider et al., 2012). The distribution of frequency shift estimated using the various three-signal compartment models showed distinct frequency shift peaks (Figure 8). The myelin frequency shift had the widest distribution of values, and frequency shift results agree with frequency shift distribution predictions based on simulations (Sati et al., 2013). The axonal (-10 to 10 Hz) and extracellular frequency shift (-5 to 15 Hz) ranges overlap to some extent, making it difficult to distinguish axonal and extracellular frequency shifts without additional information.

### Technical Considerations

Apart from myelin, both iron and protein content can also affect the signal decay (Duyn et al., 2007) in white matter.

Iron and myelin co-localize in white matter with a similar signal decay (Fukunaga et al., 2010; Denk et al., 2011). In this study, we only considered a myelin compartment and we did not account for a separate iron compartment which affects the proper estimation of myelin content (Birkl et al., 2019). In addition, signal decay is affected by fiber orientation and thereby influences the model parameters (Lee et al., 2010; Wharton and Bowtell, 2012). Furthermore, frequency shift and relaxation time are also affected by tissue, iron, and myelin compactness (Chen et al., 2013; Kor et al., 2019). Since the fibers are fairly perpendicular to B0 in the corpus callosum, we chose the corpus callosum as a test case to study the effect of tissue parameters using different modeling techniques. Extending the models to a whole-brain analysis would be very desirable but would mean that this effect needs to be included in the models, which would lead to increasing model complexity and reducing robustness further, an investigation that would be very valuable and interesting but beyond the aim of this study. The g-ratio estimated by signal compartmentalization largely depends on the proper estimation of MWF and AWF. T_1_ effects can potentially influence the calculation of signal compartment water fractions (Oh et al., 2013); however, the effect likely cancels out in the g-ratio calculation. The use of a short TR can lead to an overestimation of the MWF (Du et al., 2007). Furthermore, it is difficult to distinguish between the axonal and extracellular signal compartments based on T_2_* values (Xu et al., 2017) and similar frequency shifts (Figure 8), hence why the g-ratio might be under or overestimated depending on how axonal and extracellular compartments are decided. Skinner et al. (2007) also showed that varying echo spacing in multi-echo spin echo data will affect the estimation of MWF. However, varying echo spacing in multi-echo gradient echo data has not been studied to date. Therefore, the effect of echo spacing in MWF using multi-echo gradient echo data is still unknown. However, it has been shown that the number of echoes in multi-echo gradient echo data used to estimate the three-compartment model affects tissue parameters (Nam et al., 2015).

Tendler and Bowtell (2019) found that the frequency shift could be impacted by residual phase offsets in the GRE-MRI signal. West et al. (2019) showed that careful consideration should be made for the choice of optimization method used to generate model parameters.

## Conclusion

We investigated the utility of existing GRE-MRI signal compartment models for the characterization of the corpus callosum. We implemented existing three-signal compartment models and evaluated whether the number of parameters can be reduced while maintaining robust mapping of MWF and g-ratio. We found the reduced five parameter two-signal compartment model to be inadequate. Three-signal compartment models were able to produce comparable MWF and g-ratio maps. Based on our findings, it also appears that at least a three-signal compartment model with seven free parameters is required. Through compartment modeling, the additional estimation of compartment frequency shifts may lead to improved characterization of white matter, in turn allowing estimation of myelin loss and changes in myelination.

## Data Availability Statement

The datasets generated for this study are available on request to the corresponding author.

## Ethics Statement

The studies involving human participants were reviewed and approved by The University of Queensland. The patients/participants provided their written informed consent to participate in this study.

## Author Contributions

KT, VV, SB, and MB conceived and involved in study design, analysis, discussion and interpretation of results, and revision of manuscript. KT and VV wrote the manuscript.

## Conflict of Interest

The authors declare that the research was conducted in the absence of any commercial or financial relationships that could be construed as a potential conflict of interest.

## Footnotes

1 https://people.eecs.berkeley.edu/~chunlei.liu/software.html

2 https://mipav.cit.nih.gov
