# Supplementary material for: Influence of 7T GRE-MRI Signal Compartment Model Choice on Tissue Parameters
Source: Front Neurosci. 2020 Apr 28;14:271. doi: 10.3389/fnins.2020.00271 (PMC7206227; doi:10.3389/fnins.2020.00271)
Supplement: Supplementary file 1 [file Data_Sheet_1.pdf]

Estimated tissue parameters using different modelling techniques

Table 1 Mean and standard deviation of myelin water fraction across 10 participants obtained using 2COMP, 3COMP, SATI, NAM and THAPALIYA

Regions/methods	2COMP	3COMP	SATI	NAM	THAPALIYA
Genu	0.15 (0.07)	0.13 (0.07)	0.18 (0.11)	0.18 (0.1)	0.25 (0.07)
Rostral	0.18 (0.09)	0.23 (0.05)	0.23 (0.03)	0.23 (0.03)	0.27 (0.02)
Anterior mid-body	0.29 (0.07)	0.26 (0.07)	0.23 (0.04)	0.23 (0.04)	0.27 (0.06)
Posterior mid-body	0.3 (0.07)	0.27 (0.08)	0.27 (0.04)	0.25 (0.04)	0.29 (0.07)
Isthmus	0.23 (0.06)	0.24 (0.09)	0.24 (0.07)	0.24 (0.07)	0.3 (0.04)
Splenium1	0.21 (0.09)	0.22 (0.08)	0.25 (0.06)	0.27 (0.06)	0.29 (0.07)
Splenium2	0.21 (0.08)	0.27 (0.08)	0.27 (0.09)	0.28 (0.08)	0.3 (0.09)

Table 2 Mean and standard deviation of myelin frequency shift across 10 participants obtained using 3COMP, SATI, and NAM model

Regions/methods	3COMP	SATI	NAM
Genu	48.53 (25.75)	26.03 (21.03)	45.21 (22.13)
Rostral	33.51 (6.16)	33.82 (5.02)	33.54 (5.38)
Anterior mid-body	31.27 (8.29)	37.07 (13.73)	36.46 (13.79)
Posterior mid-body	28.09 (7.33)	27.24 (6.77)	28.66 (6.21)
Isthmus	27.95 (7.5)	28.02 (7.68)	28.57 (7.32)
Splenium1	23.59 (9.48)	22.16 (8.58)	20.00 (9.81)
Splenium2	21.31 (11.16)	23.20 (9.51)	22.31 (9.22)

Table 3 Mean and standard deviation of axonal water fraction across 10 participants obtained using 3COMP, SATI, NAM and THAPALIYA model

Regions/methods	*3COMP*	*SATI*	*NAM*	*THAPALIYA*
Genu	*0.32 (0.11)*	*0.44 (0.12)*	*0.34 (0.1)*	*0.37 (0.08)*
Rostral	*0.44 (0.12)*	*0.39 (0.09)*	*0.42 (0.05)*	*0.47 (0.06)*
Anterior mid-body	*0.52 (0.06)*	*0.43 (0.06)*	*0.40 (0.07)*	*0.49 (0.1)*
Posterior mid-body	*0.54 (0.09)*	*0.45 (0.06)*	*0.45 (0.06)*	*0.47 (0.06)*
Isthmus	*0.50 (0.09)*	*0.42 (0.16)*	*0.44 (0.16)*	*0.45 (0.08)*
Splenium1	*0.43 (0.12)*	*0.36 (0.13)*	*0.33 (0.11)*	*0.44 (0.05)*
Splenium2	*0.37 (0.13)*	*0.34 (0.22)*	*0.32 (0.12)*	*0.44 (0.11)*

Table 4 Mean and standard deviation () of axonal frequency shift across 10 participants obtained using 3COMP, SATI, and NAM model.

Regions/methods	*3COMP*	*SATI*	*NAM*
Genu	*-6.86 (3.49)*	*-5.27 (2.06)*	*2.67 (5.44)*
Rostral	*-1.27 (1.68)*	*-1.47 (1.37)*	*4.68 (4.92)*
Anterior mid-body	*0.59 (1.11)*	*0.13 (1.16)*	*2.83 (5.43)*
Posterior mid-body	*-0.03 (0.98)*	*-0.52 (0.94)*	*0.32 (3.17)*
Isthmus	*-1.76 (1.58)*	*-2.10 (1.81)*	*1.66 (4.91)*
Splenium1	*-5.56 (2.35)*	*-6.40 (2.35)*	*-6.63 (2.13)*
Splenium2	*-7.15 (2.79)*	*-7.62 (3.5)*	*-6.58 (4.52)*

Table 5 Mean and standard deviation () of extracellular water fraction across 10 participants obtained using 3COMP, SATI, NAM and THAPALIYA model

Regions/methods	*3COMP*	*SATI*	*NAM*	*THAPALIYA*

Genu	*0.55 (0.11)*	*0.38 (0.1)*	*0.48 (0.14)*	*0.38 (0.1)*
Rostral	*0.33 (0.13)*	*0.38 (0.08)*	*0.35 (0.06)*	*0.26 (0.05)*
Anterior mid-body	*0.22 (0.02)*	*0.34 (0.07)*	*0.36 (0.05)*	*0.24 (0.11)*
Posterior mid-body	*0.20 (0.03)*	*0.28 (0.05)*	*0.30 (0.06)*	*0.24 (0.05)*
Isthmus	*0.26 (0.13)*	*0.34 (0.13)*	*0.32 (0.12)*	*0.25 (0.1)*
Splenium1	*0.35 (0.15)*	*0.40 (0.11)*	*0.40 (0.12)*	*0.28 (0.05)*
Splenium2	*0.35 (0.16)*	*0.39 (0.2)*	*0.40 (0.09)*	*0.26 (0.16)*

Table 6 Mean and standard deviation of extracellular frequency shift across 10 participants obtained using 3COMP, SATI, and NAM model.

Regions/methods	*3COMP*	*SATI*	*NAM*
Genu	*2.63 (2.62)*	*4.37 (3.09)*	*-4.26 (3.55)*
Rostral	*8.47 (1.6)*	*7.64 (1.39)*	*0.89 (4.2)*
Anterior mid-body	*11.98 (1.24)*	*10.69 (1.63)*	*7.25 (4.7)*
Posterior mid-body	*11.20 (0.83)*	*9.65 (1.54)*	*8.62 (3.2)*
Isthmus	*7.26 (2.76)*	*5.29 (3.44)*	*0.71 (4.26)*
Splenium1	*2.03 (2.23)*	*1.56 (1.6)*	*1.63 (1.5)*
Splenium2	*1.32 (2)*	*0.88 (2.58)*	*0.06 (1.69)*

Table 7 Mean and standard deviation of error rate (%) across 10 participants obtained using 2COMP, 3COMP, SATI, NAM and THAPALIYA model

Regions/methods	*2COMP*	*3COMP*	*SATI*	*NAM*	*THAPALIYA*
Genu	*14.61 (10.64)*	*8.84 (7.68)*	*7.08 (6.79)*	*7.45 (6.75)*	*4.56 (4.08)*
Rostral	*8.95 (3.56)*	*3.93 (1.58)*	*3.77 (1.48)*	*3.79 (1.5)*	*2.86 (1)*
Anterior mid-body	*12.69 (2.65)*	*3.68 (0.86)*	*3.44 (0.84)*	*3.39 (0.78)*	*2.72 (0.74)*
Posterior mid-body	*11.38 (3.57)*	*3.11 (1.37)*	*2.94 (1.32)*	*2.94 (1.38)*	*2.61 (0.85)*
Isthmus	*7.16 (3.52)*	*2.72 (1.13)*	*2.38 (1.18)*	*2.33 (1.19)*	*1.99 (0.89)*
Splenium1	*6.42 (3.25)*	*3.09 (1.98)*	*2.56 (1.7)*	*2.64 (1.72)*	*1.75 (1.17)*
Splenium2	*6.70 (2.76)*	*3.84 (1.55)*	*3.22 (1.54)*	*3.29 (1.51)*	*2.33 (1.18)*
